# A conserved strategy for structure change and energy transduction in Hsp104 and other AAA+ protein motors

**DOI:** 10.1016/j.jbc.2021.101066

**Published:** 2021-08-09

**Authors:** Xiang Ye, Leland Mayne, S. Walter Englander

**Affiliations:** 1 Department of Biochemistry and Biophysics, Texas A&M University, College Station, Texas, USA; 2 Department of Biochemistry and Biophysics and Johnson Research Foundation, Perelman School of Medicine, University of Pennsylvania, Philadelphia, Pennsylvania, USA

**Keywords:** hydrogen exchange mass spectrometry, cryo-electron microscopy, optical tweezers, AAA+ proteins, molecular motor, AAA+, ATPases associated with diverse cellular Activities, cryo-EM, cryogenic electron microscopy, HX MS, hydrogen-deuterium exchange analyzed by mass spectrometry, OT, optical tweezer

## Abstract

The superfamily of massively large AAA+ protein molecular machines functions to convert the chemical energy of cytosolic ATP into physicomechanical form and use it to perform an extraordinary number of physical operations on proteins, nucleic acids, and membrane systems. Cryo-EM studies now reveal some aspects of substrate handling at high resolution, but the broader interpretation of AAA+ functional properties is still opaque. This paper integrates recent hydrogen exchange results for the typical AAA+ protein Hsp104 with prior information on several near and distantly related others. The analysis points to a widely conserved functional strategy. Hsp104 cycles through a long-lived loosely-structured energy-input “open” state that releases spent ADP and rebinds cytosolic ATP. ATP-binding energy is transduced by allosteric structure change to poise the protein at a high energy level in a more tightly structured “closed” state. The briefly occupied energy-output closed state binds substrate strongly and is catalytically active. ATP hydrolysis permits energetically downhill structural relaxation, which is coupled to drive energy-requiring substrate processing. Other AAA+ proteins appear to cycle through states that are analogous functionally if not in structural detail. These results revise the current model for AAA+ function, explain the structural basis of single-molecule optical tweezer kinetic phases, identify the separate energetic roles of ATP binding and hydrolysis, and specify a sequence of structural and energetic events that carry AAA+ proteins unidirectionally around a functional cycle to propel their diverse physical tasks.

AAA+ proteins are designed to convert the chemical energy packaged in cytosolic ATP into physicomechanical form and use it to perform an extraordinary number of energy-requiring physical operations throughout all of biology (1, 2, 3, 4, 5, 6, 7, 8). The superfamily of AAA+ protein molecular machines, with over 200 members in humans alone (9), is defined by the possession of one or more canonical AAA+ domains (ATPases associated with diverse cellular activities). Their myriad functions range from homeostasis and regulation in the world of proteins, through DNA replication and recombination, and ribosomal RNA processing, to organelle biogenesis and membrane fusion. The large AAA+ literature runs to thousands of publications. This paper is not intended as a literature review. Rather, it draws on new information obtained in recent hydrogen exchange studies (10, 11) to broaden current interpretation of experimental results in the AAA+ literature and to search for commonalities in AAA+ mechanism. The analysis identifies the essential roles of a loosely structured open state that has been known before but has been largely ignored, and it uncovers a functional strategy that interlocks structure change and energy transduction cycles. The same strategy appears to be widely conserved among AAA+ protein motors.

Advances in electron microscopic resolution and analysis now add to earlier crystallographic results (12, 13) to show what an increasing number of AAA+ molecular machines look like and suggest how they might work. Figure 1 shows some typical examples. Fungal Hsp104 functions in *Saccharomyces cerevisiae* to dissociate aggregated proteins and to foster and disrupt prions such as Sup35 on demand (14, 15). Bacterial Lon protease from *Yersinia pestis* is involved in protein unfolding and proteolytic degradation (16). Both proteins are hexameric and adopt similar whole molecule configurations, called open and closed states, with the protomers arrayed around a central channel in a relatively planar right-handed spiral in the ATP-bound closed state and a more steep left-handed spiral in the apo or ADP-bound open state. They both use their pore loops to seize substrate proteins and forcefully thread them into and through their axial channel. These features are common to AAA+ proteins but differences are also widespread. For example, Hsp104 has two canonical nucleotide-binding domains (NBD1 and NBD2) with an inserted middle domain (MD) in NBD1. It also features an extended state that resembles its closed state, leading to a stepwise so-called hand-over-hand model for substrate translocation that pictures an ATPase-driven interchange between the closed and extended forms. Lon has one NBD, a covalently attached C-terminal protease domain, no MD, and no observed extended state. Thus, as for the wide range of other AAA+ proteins, Hsp104 and Lon protease display significant structural differences, but they retain similarities that suggest similar functional strategies.

**Figure 1:**
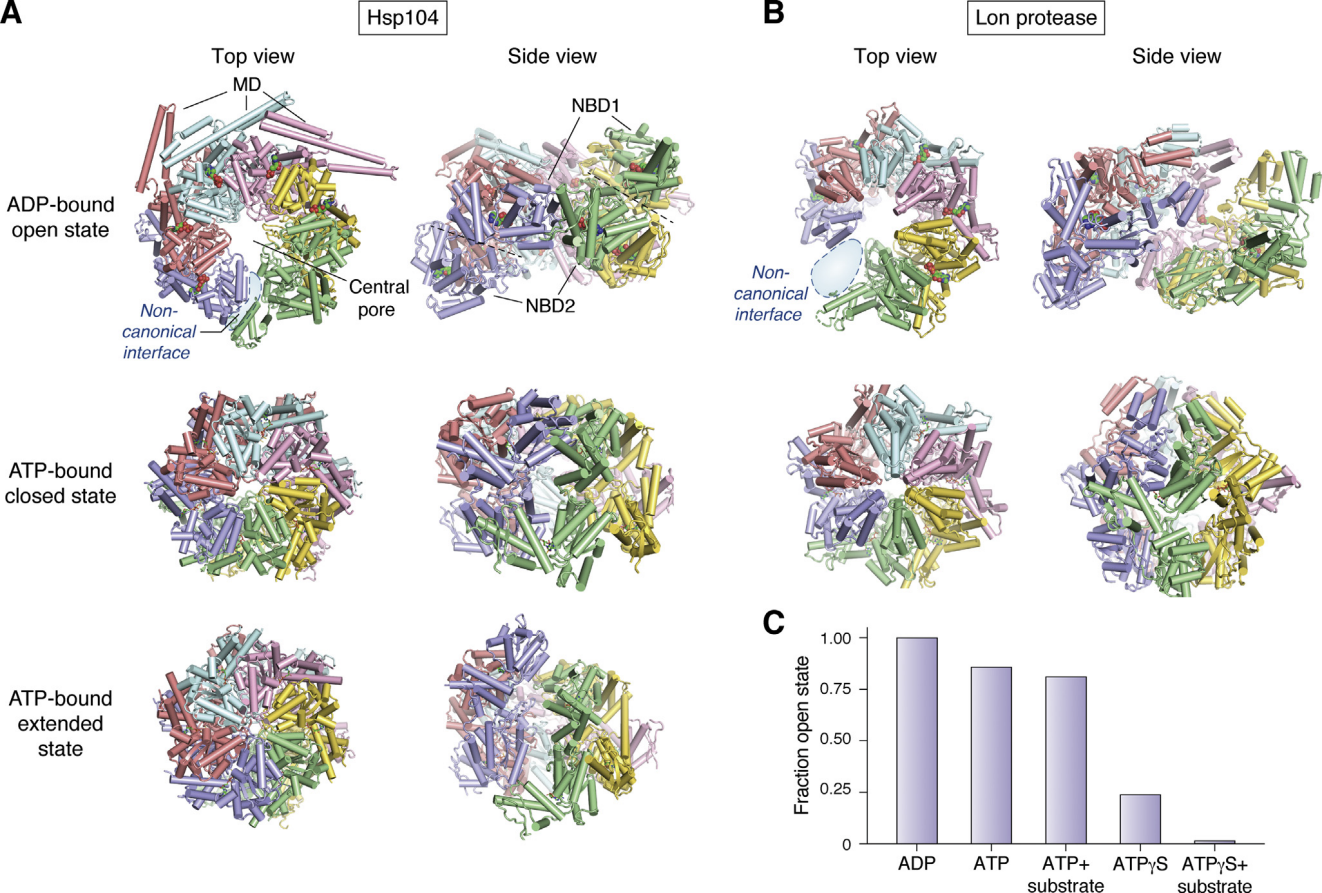
**Cryo-EM structures of Hsp104 and Lon protease**. *A*, Hsp104 in the ADP-bound open state (PDB 5VY8), the ATPγS-bound closed state (PDB 5VJH), and the extended state (PDB 5VYA) (14, 15). The six protomers are in different colors. The two nucleotide-binding domains (NBD1 and NBD 2, *upper* and *lower*) can be seen in the more strongly spiraled open state side view, which also has one mismatched noncanonical interprotomer interface. The loosely structured (11) N- and C-terminal domains were not resolved and the MDs were resolved only for three protomers in the open state (*top left*). In the open state, neighboring MDs link noncovalently to suppress the dissociation of ADP shown as red beads, extend the open state lifetime, and downregulate activity. *B*, Lon protease shows similar open (PDB 6V11) and closed (PDB 6ON2) states but also a covalently attached C-terminal protease domain and no extended state or MD (16). Significantly, the Lon open state under turnover conditions is seen to be fully occupied by ADP, as for Hsp104 due to the slow release from the open state of retained product ADP following multiple ATPase reactions in the closed state. *C*, fractional occupation of the Hsp104 open state ranging from 100% in the static ADP-bound open state, to ∼85% during active turnover, to a low level in the nearly static ATPγS-bound closed state condition. The active turnover condition (ATP), with a dominant fraction of open/NEx state occupation, matches the HX MS and OT results described here. The current hand-over-hand sequential translocation model, with little role for the open state, is based on cryo-EM images statically trapped in the closed/SPr state by bound poorly hydrolyzable ATPγS.

Hydrogen-deuterium exchange (HX) results offer new insight. Following a long history of development in both measurement (17, 18, 19, 20, 21, 22) and interpretation (23, 24, 25, 26, 27, 28), HX analyzed by fragment-separation mass spectrometry (HX MS) now provides a powerful tool for the study of molecular biophysics. HX is able to report on structure, structure change, internal dynamics, energetics, and functional interactions, all at nearly amino acid resolution (25, 27, 29, 30). In recent work, we used HX MS technology (Fig. 2) to measure main chain amide HX throughout the model 0.6 MDa Hsp104 AAA+ protein (10, 11). We obtained complete data over the entire HX timescale (10 ms to 20 h) at near site resolution for Hsp104 in its different structural and nucleotide-bound states under both static and active-turnover conditions (10, 11). The results provide access to the two main drivers of Hsp104 function, ATP energetics and protein structure change, and reveal the local and global structural, energetic, and functional effects of nucleotide ligands, substrates, and mutations during active turnover in physiological solution.

**Figure 2:**
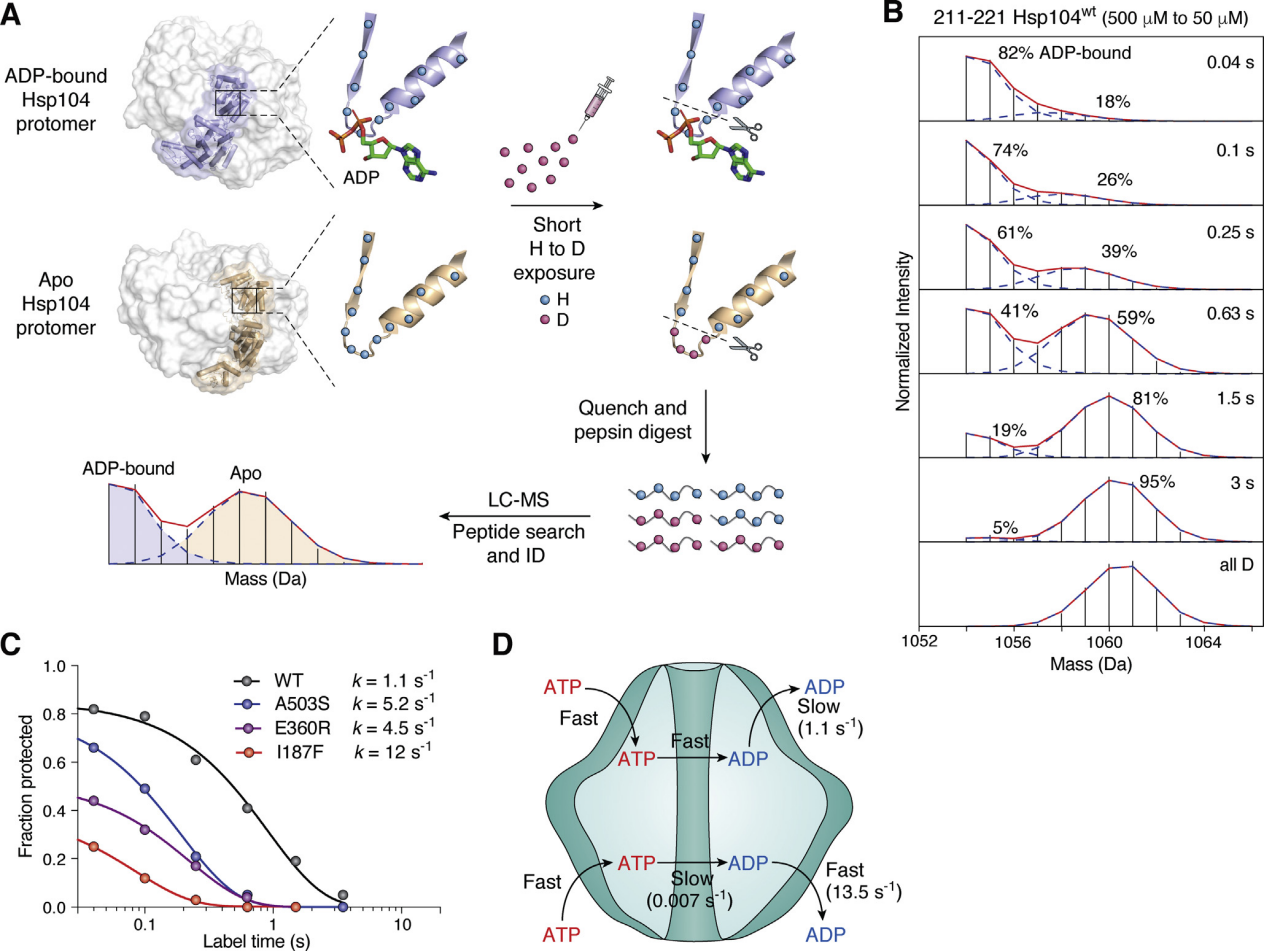
**HX MS analysis and the measurement of ADP dissociation.***A*, in previous work (10, 11), Hsp104 in physiological solution in various static conditions or during functioning was exposed to amide H to D exchange in D_2_O solvent. Timed samples were quenched into a slow HX condition and injected into a flow system for online proteolysis into many peptides (∼250). The peptide mix was roughly resolved by fast LC and electrospray-injected into the mass spectrometer for further separation and analysis of their D-content. A bimodal mass spectrum for the nucleotide-binding Walker A segment of Hsp104 NBD1 (residue 211–221) is shown at one time point during an ADP dissociation experiment (10). The Walker A segment is protected when ADP is bound but when ADP dissociates (Apo) it deuterates immediately (mass increases) before rebinding can occur. Similar results for the many peptides measure the site-resolved structural, energetic, and functional effects of nucleotide ligands, substrates, and mutations all through the protein (11, 22, 57). *B*, Hsp104 initially in high ADP (500 μM; open state) was diluted into D_2_O at t = 0. The two isotopic MS envelopes represent the still ADP-bound HX-protected population (lighter) and the increasing fraction that has experienced ADP dissociation and D-labeling (heavier) during the H to D exchange period. Timed samples were analyzed to measure the rate of ADP dissociation. *C*, the time course of ADP dissociation from WT Hsp104 and three mutationally potentiated variants, each fit with a single exponential decay. Fraction protected refers to the decreasing fraction of Walker A sites still protected from HX due to bound ADP, measured as in *panel B*. The exponential decay indicates that the multiple protomers dissociate ADP independently, all with the same rate constant in the loosely structured open state. The initial value measures the equilibrium (K_D_) that exists between free ADP and ADP-bound protomers at the beginning of the measurement. *D*, the Hsp104 ATPase cycle with WT parameters. ADP release from NBD1 (upper ring) is the rate-limiting step (∼1 s^−1^) in active turnover. In NBD2, ATP hydrolysis is far slower (<0.01 s^−1^).

Here we integrate this new information with prior cryo-EM and single-molecule optical tweezer (OT) studies. We consider the structural and energetic strategies that AAA+ proteins employ, despite their structural differences, to accomplish their functional goals. The results of this analysis reinterpret the current cryo-EM model, explain the structural basis of single-molecule OT results (Fig. 3), define the energetic roles of ATP binding and hydrolysis, and unite structure change and ATP-based energetic functions in a novel mechanochemical cycle. We find that active Hsp104 travels unidirectionally around a structure-change cycle through a long-lived open state as well as through the briefly occupied closed and extended states. At the energy-input open-state stage, ADP dissociation and ATP rebinding drive the energetically uphill open to closed transition, priming the physical trigger. At the output closed-state stage, ATP hydrolysis releases the trigger and allows downhill relaxation, which couples to energy-requiring forceful substrate translocation. We organize here the evidence for this functional strategy and consider whether it might be evolutionarily conserved.

**Figure 3:**
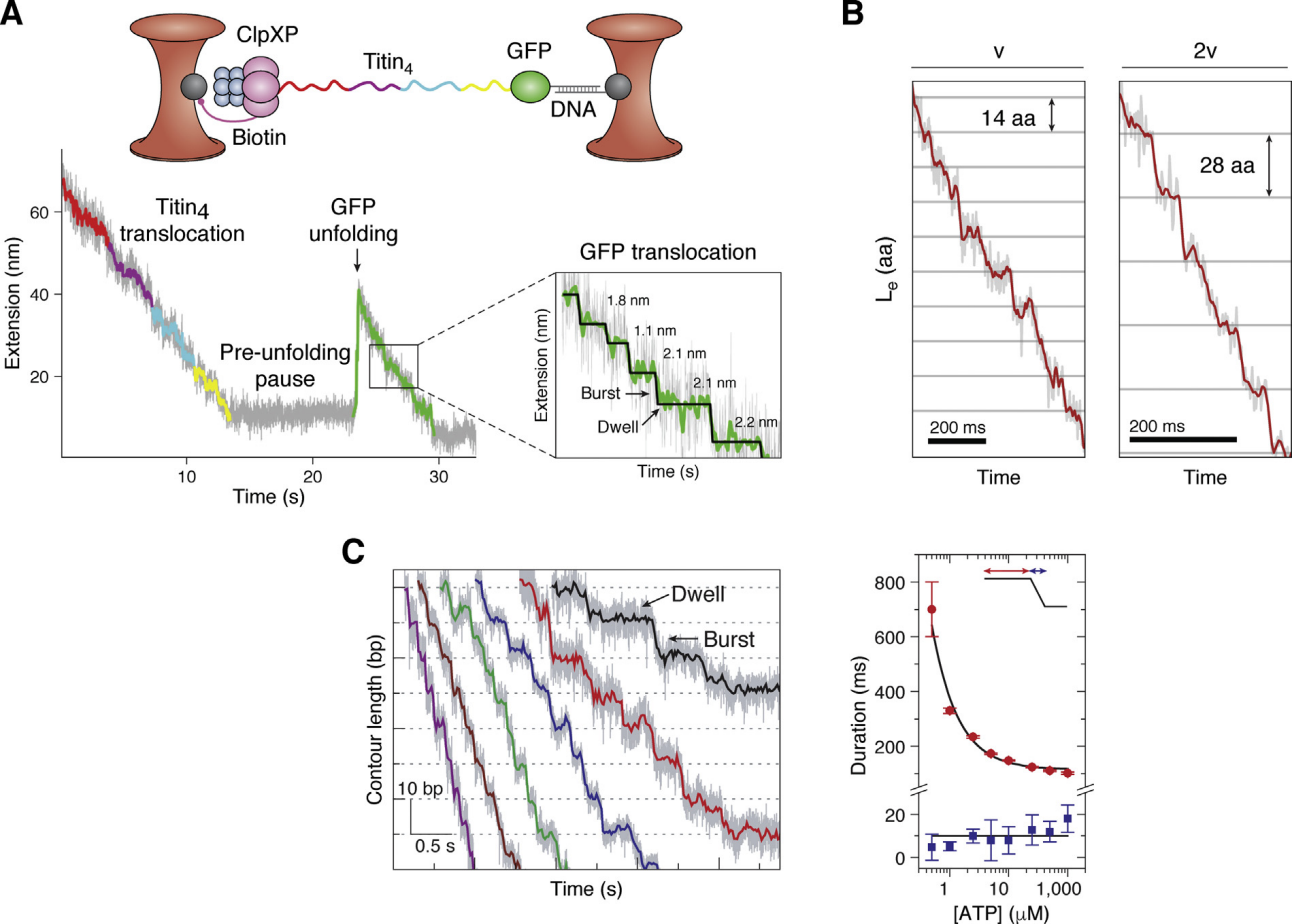
**Single-molecule dual optical tweezer analysis.***A*, example OT setup and data for ClpXP. ClpXP is immobilized on a micron-sized bead trapped in a laser beam, and the substrate is bound to the other bead (46). In this experiment, the substrate had four unfolded titin I27 domains (titin_4_) C-terminally fused to a green fluorescent protein (GFP). The OT trajectory registers the translocation of preunfolded titin, a pause in the face of GFP resistance, GFP unfolding requiring prior force buildup, and unfolded GFP translocation. Expansion: Noise-filtered segment of unfolded GFP translocation displaying variable dwells (*horizontals*) and bursts (*verticals*). *B*, OT translocation trajectories of maltose binding protein (MBP) by a potentiated variant of ClpB (Y503D) (49). MBP tethered within a dual OT was unfolded, relaxed to a low force that prohibits refolding, and exposed to ClpB and ATP in solution. Translocation trajectories for a single strand (speed v, 14 aa/burst) or two strands of an unfolded MBP loop (speed 2v, 28 aa/burst) are shown, translated to change in polypeptide contour length (*L*_*e*_). *C*, OT results for the translocation of double-stranded DNA by the ϕ29 phage DNA-packaging motor (50). The motor complex and its dsDNA substrate are tethered between two laser-trapped beads under tension. *Left*, dwell/burst traces as a function of free [ATP] decreasing left to right (250 μM to 5 μM). *Right*, duration of the dwell phase (*red*) and the burst phase (*blue*) as a function of free [ATP] (47). At very low [ATP], the dwell lifetime (open/NEx state) is lengthened, evidently due to the slow rebinding of sufficient ATP, necessary to shift the open to closed equilibrium and terminate each dwell phase. Even at high [ATP] the dwell phase duration, limited apparently by ADP dissociation (<10 s^−1^), accounts for the large fraction of total cycle time. Figures are reproduced with permission from (46) (*A*) (49), (*B*), and (50) (*C*).

Given that the detailed structures of AAA+ proteins differ in response to their different tasks and circumstances, we will refer to known open and closed states in Hsp104 as such and refer to hypothetical not yet known but apparently analogous states in other AAA+ proteins in terms of their functional properties, defined here as the open nucleotide exchange state (open/NEx) and the closed substrate processing state (closed/SPr).

## Hsp104 cycles through the open and closed states

In earlier work (10, 11), we tested the ability of HX MS to detect in physiological solution the whole hexamer closed, extended, and open states of Hsp104 that had been described by cryo-EM (Fig. 1) (14, 15). Under static conditions and during active turnover, HX MS was able to recognize and characterize the open state and also the closed/extended states, which however we could not separately distinguish. The more loosely structured open state, with or without bound ADP, is marked by faster HX, for example, at the substrate-binding pore loops, and at the one noncanonical interprotomer interface where the top and bottom of the spiraled hexamer rejoin, and also by poor ability to retain substrate during processing. In the closed/extended states driven by ATP binding, the six protomers become equivalent, HX of the pore loops is 100-fold slower, and substrate is more effectively retained.

An influential observation was that the site-resolved ADP dissociation rate, unavailable to other methods, could be measured by HX MS (10) (Fig. 2*A*). The rate of ADP dissociation from NBD1 in the static open state, 1.1 s^−1^ (Fig. 2*B*), is nearly equal to the ATPase rate during active turnover (10). Thus ADP dissociation from NBD1 in the open state appears to be the rate-limiting step in the functional cycle. The effect is indirect. Slow ADP dissociation from the open state rate-limits ATP rebinding, which drives the protein to its active closed state where the ATPase reaction and substrate processing occur. In agreement, the acceleration of ADP dissociation in mutationally potentiated Hsp104 variants by factors up to 10-fold (Fig. 2*C*) is matched by the increase in their ATPase rates and in their processing power (10). In NBD2, the ATPase reaction contributes negligibly to measured ATP turnover because it is 150-fold slower than the NBD1 rate (Fig. 2*D*) (31).

These results open a new window on Hsp104 function. The well-known fact that ATP hydrolysis and substrate translocation occur in the closed state motivates current functional models. However, the observation that the dissociation of product ADP occurs from the open state and rate-limits ATPase activity in the closed state ensures, surprisingly, that active Hsp104 must cycle through both states. The slow rate-limiting dissociation of ADP from the open state during turnover ensures that open state occupation is long-lived. Other HX MS results (10, 11) confirm that active Hsp104 cycles through both the open and closed states and spends by far the largest fraction of cycle time, roughly 90%, in the open state (10). This value comes from measurement of the HX rate of the substrate-binding pore loops, which is faster in the open state by 100-fold and thus can measure the fraction of time spent in each state during cycling. Cryo-EM and single-molecule OT results agree when they are properly considered, as described below.

## Essential roles of the open state in Hsp104 function

HX MS results show that the Hsp104 open state is responsible for the nucleotide exchange function and also for the regulation of its activity (10). The preponderant time spent in the open state during active turnover largely determines the time required for each functional cycle, therefore the number of cycles s^−1^ and the power output (ATPase s^−1^). The amount of time spent in the loosely structured open state also modulates the loss of substrate protein during processing, which can be quite large. During active turnover, the steady-state level of substrate-bound WT Hsp104 is only ∼5%, and it increases about as expected in potentiated mutants with accelerated ADP off rate and shortened open state lifetime (10).

The four-helix MD of Hsp104 regulates its activity by tuning its open state lifetime (10). The MD is placed across the Hsp104 NBD1 interprotomer region that harbors the nucleotide-binding site (Fig. 1*A*) and suppresses the dynamics that allow nucleotide entry and exit. The slowing of ADP release extends the open state lifetime, therefore the time needed for each cycle, and it increases the loss of substrate and so downregulates the potential cytotoxicity of this otherwise powerful protein unfoldase. In the opposite direction, ADP dissociation can be accelerated and activity increased by mutational potentiation (Fig. 2*C*) and by interaction of the cochaperone Hsp70/40 system with the exposed regulatory MD (10, 32, 33, 34). Similarly, as described below, other AAA+ proteins when potentiated as for ClpB, or without an MD as for ClpX and the ϕ29 motor, have shorter open state lifetime, measured by OT dwell time, and they retain substrate more effectively.

In summary, HX MS proved to be able to recognize the open and closed states of Hsp104 defined structurally by cryo-EM. HX MS showed that Hsp104 during active turnover cycles through the open state as well as the closed state. The loosely structured open state is responsible for sufficiently fast dissociation of ADP produced in prior ATPase reactions and also for the rebinding of fresh ATP (as described below), resetting the protein for the next round of substrate processing in the closed state. Nevertheless, the ADP off rate is still relatively slow (∼1 s^−1^). This dictates that the open state not only recurs repeatedly during turnover but that its lifetime is so long that it dominates the time required for each closed state/open state cycle. Manipulation of the long open state lifetime underpins Hsp104 molecular regulation.

## Cryo-EM confirms the major occupation of the open state during active cycling

When AAA+ proteins are held in a nearly static noncycling condition by the binding of an inactive ATP analogue (ATPγS) or by ATPase-inactivating mutations, they are stalled in substrate processing conformations. Cryo-EM of Hsp104 then detects mainly the population of closed and extended states (Fig. 1*C*) with protein substrate held unfolded in the axial channel spaced at two amino acids per protomer (14). From similarly selective images of various AAA+ proteins, many authors (16, 35, 36, 37, 38, 39, 40, 41, 42, 43) have inferred an appealing model for substrate processing, based on the distribution through the spiraled protomers of not-yet-hydrolyzed ATPs and spent ADPs. Unfolded substrate is pictured to be continuously translocated in a stepwise hand-over-hand manner through the axial channel in sequential two-amino-acid steps. Each step is pictured to be propelled structurally by a closed state–extended state interchange and energetically by ATP hydrolysis (14, 15).

While the trapping approach effectively focuses on the substrate processing state, it is blind to populations that may manage other parts of the cycle. Further limiting a more comprehensive view, the remarkable success of high-resolution cryo-EM has led to a narrowed focus on the finer details of substrate processing. When cryo-EM images of Hsp104 are obtained during active cycling so that all of the participating protein conformations are observed, a dominant steady-state open-state fraction is seen, ∼85% of the population (Fig. 1*C*), in agreement with HX MS. The same is true for Lon protease (Fig. 1*B*).

It has also been noted (44, 45, 46, 47, 48) that the strictly sequential hand-over-hand model alone is not consistent with some irregularities in measured substrate translocation. Translocation steps in the burst phase of optical trap experiments can be larger than two residues (7, 48, 49, 50) (Fig. 3), not strictly sequential, and not blocked by intervening ATPase-inactive protomers. These unexpected behaviors might be explained by the variable ATP-rebinding property of the open/NEx state, as described below.

In summary, it appears that the current sequential processing model based on cryo-EM images is incomplete. The experimental design of cryo-EM studies has almost always focused selectively on substrate processing subpopulations trapped in the static closed/SPr state. The synthesis of HX MS and cryo-EM results point to a more complete functional model in which the briefly occupied closed/SPr state alternates with a long-lived open/NEx state population, with substrate processing and ATP functions distributed between them.

## Optical tweezers burst and dwell phases represent the closed/SPr and open/NEx states

Single-molecule OT experiments resolve the structural cycling of AAA+ proteins during active turnover into distinct kinetic phases. Figure 3 shows examples. OT substrate pulling trajectories display a series of burst phases (verticals) that represent the changing distance between anchoring beads, alternating with resting dwell phases (horizontals). The brief burst phase (tens of msec) directly measures the added or decreased extension of unfolded polypeptide or dsDNA due to substrate translocation. The lengthy dwell phase (hundreds of msec) measures a regularly repeating halt in substrate translocation. It is striking that similar kinetic phases with similar amplitudes and duration are found for the distantly related single NBD heterohexameric ClpX (7, 44, 45, 46, 47, 48) (Fig. 3*A*), the double NBD homohexameric ClpB (49) (Fig. 3*B*), and even for the single NBD pentameric ϕ29 phage DNA-packaging protein (50, 51, 52) (Fig. 3*C*). These similarities suggest that these AAA+ proteins share a common cycling strategy.

Cryo-EM results show that active substrate translocation occurs in the closed state. The OT observation that substrate translocation occurs in the burst phase associates the OT kinetic burst phase with the cryo-EM structural open state. The origin of the lengthy OT dwell phase is not known. The newly documented involvement of the open/NEx state in the active cycle (11) suggests that it may account for the dwell phase. Several lines of evidence support this view. Cryo-EM images of Hsp104 taken during active turnover rather than when stalled in the closed/SPr state display a dominant ∼85% steady-state population of the open state (Fig. 1*C*). HX MS results for Hsp104 during active turnover affirm that it spends the great majority of its cycling time, roughly 90%, in the open state (10, 11). OT results for Hsp104 are not yet available, but they are for the close homolog ClpB (49). The ClpB dwell phase (Fig. 3*B*) appears to account for a significantly smaller fraction of its cycle time, ∼50%, than is seen for Hsp104 by cryo-EM and HX MS, but that estimate greatly increases and falls into the same ∼85% grouping when it is considered that the ClpB OT data were obtained for a potentiated variant, which shortens the open state period by 5-fold (49). These observations associate the lengthy OT dwell phase with the long-lived Hsp104 open/NEx state. OT results in Figure 3 for other proteins show that their dwell phase similarly accounts for a large majority of the cycle time, suggesting a similar open/NEx state relationship. A functional test is even more definitive. ADP dissociation and ATP rebinding have been found to occur in the kinetic dwell phase of ClpX and also in the ϕ29 motor (46, 50, 51, 52). HX MS showed that these same functions occur in the open state of Hsp104 (10).

This view has explanatory power. For example, Figure 3*C* shows that the mean dwell phase lifetime of the ϕ29 motor lengthens at very low [ATP] (50). In the present view, this occurs because, at very low [ATP], the binding of multiple ATPs (four estimated (50)) becomes rate-limiting, *i.e.*, even slower than the previously rate-limiting ADP off rate. The same [ATP] dependence was not seen for ClpXP (47), perhaps because sufficiently low [ATP] could not be explored or its ATP on rate is faster.

## Minimizing time by grouping the slow steps

Detailed OT results provide additional information. It appears that ClpB (49), ClpX (7, 48), and the ϕ29 motor (50) can execute several rapidly successive ATPase reactions in the brief burst phase period without intervening slow ADP release. ClpB advances its substrate by ∼14 amino acids in each brief burst phase run, which would require six steps with the interprotomer two amino acid spacing. ClpX and the ϕ29 motor manage up to four steps in each burst phase, either separately resolved or as gauged by comparing the measured burst phase length change with the interprotomer substrate spacing. These multiple steps, each one driven by an ATPase reaction (48), occur within the few tens of msec of the burst phase. Accompanying ADP dissociation from the closed state would require far more time. Thus it appears that several consecutive ATPase reactions can occur in the brief closed/SPr burst phase state without intervening ADP release. Evidently, product ADP is retained pending transition to the long-lived open/NEx dwell phase state where much more efficient collective ADP release can occur.

Reliance on the looser open/NEx state for nucleotide exchange is advantageous and perhaps even essential. Although ADP release is intrinsically slow even from the loose open/NEx state, it is much faster than could be obtained from the more tightly closed active site. Thus ADP release from the open state leads to a shortened overall cycle time. The benefit is amplified when several ATPase reactions are grouped. The collective release of several ADPs from the open state, all with the same independent rate constant (see single exponential kinetics in Fig. 2*C*), will take little more than the time for one and much less time than would be required for sequential one-at-a-time release from the closed state after each ATPase reaction. Both of these factors, a faster cycle time and more steps per cycle, minimize the negative impact of the intrinsically slow ADP release step and thus act to multiply productivity and output power.

The role of the open state in the active cycle raises an interesting question. How is substrate retained during cycling through the open state? In fact the open state of WT Hsp104 in free solution with no back force retains substrate poorly (10). Mutationally potentiated Hsp104 (10) does so somewhat better. Potentiated ClpB (49) and the other proteins in Figure 3 without an MD manage to retain substrate even under strong force conditions. Information available does not distinguish whether this capability represents intrinsic protein to protein differences or an adaptation to force or, most likely, simply their much shorter open state lifetime (∼1/10).

In summary, OT dwell and burst phases observed for these AAA+ proteins correspond, kinetically and functionally if not in structural detail, to states analogous to the open and closed states seen by HX MS and cryo-EM for Hsp104. All three methodologies agree. Hsp104 and distantly related AAA+ proteins cycle through a long-lived open-like stage as well as a brief closed-like stage with the two different stages responsible for different functions. It appears that the advantages provided by the alternating two-stage strategy have led to its evolutionary adoption in many AAA+ proteins.

## Uphill and down by ATP binding and hydrolysis

The energy required for forceful substrate processing by Hsp104 and other AAA+ proteins must be derived from either ATP binding or hydrolysis, but this fundamental question is unresolved and the energetic mechanism more broadly is unknown. How is the chemical energy of cytosolic ATP transduced into mechanical form and then mobilized to power the wide variety of AAA+ physical tasks?

HX MS results illuminate the functional energetics. The Hsp104 hexamer left to itself in a nucleotide-free condition must adopt its lowest free energy state. It is the loosely structured open state (11, 14). The binding of added ADP further stabilizes that state. During active turnover, when Hsp104 slowly off-loads ADP from the open state (k_off_ ∼ 1 s^−1^; Fig. 2), it will be promptly replaced by ATP (k_on_ ≥ 10 s^−1^ when [ATP] ≥ 1 mM) (10, 53). Cryo-EM and HX MS show that ATP binding drives Hsp104 from the open to the closed state (11, 14). (Substrate binding also favors the closed state.) The transition to the closed state elevates the protein moiety itself and suspends it in a metastable higher energy condition. The higher-energy closed state is adopted because the overall reaction, very favorable ATP binding to the open state plus the unfavorable open to closed allosteric structure change, is energetically dowhill.

The ATP-binding sites of all AAA+ proteins are enclosed within protomer interfaces. ATP and ATPγS drive the open/NEx to closed/SPr transition but ADP and AMPPNP, which differ only at the γ-PO_4_ position, do not (10, 11, 14, 15). These observations suggest that ATP binding imposes some degree of local strain that disfavors the open state in a way that is sensitive to detailed γ-PO_4_ stereochemistry. A consequence is that the following ATPase reaction in the active closed state, by cleaving the terminal phosphate of one or more ATPs, releases the strain and triggers thermodynamically downhill protein relaxation. The favorable relaxation can then be coupled to drive energy-requiring substrate translocation. If so, the force generating power stroke will (misleadingly) appear to be the permissive γ-PO_4_ release, as has been observed (46, 47). Additionally, since hydrolysis and phosphate release occur later in the closed state, this observation is against a direct contribution to substrate translocation due to earlier ATP binding in the open state or the resulting large scale open to closed transition itself, which seem to be the only possible alternatives for power-supplying steps.

In summary, the loosely structured open/NEx state is responsible for ADP release and ATP rebinding. The favorable binding of concentrated solvent ATP drives the uphill open/NEx to closed/SPr transition. Later ATP hydrolysis and γ-PO_4_ release permits rather than drives passive downhill protein relaxation, which can be coupled to substrate processing.

## The functional energy/structure-change cycle

**Figure 4:**
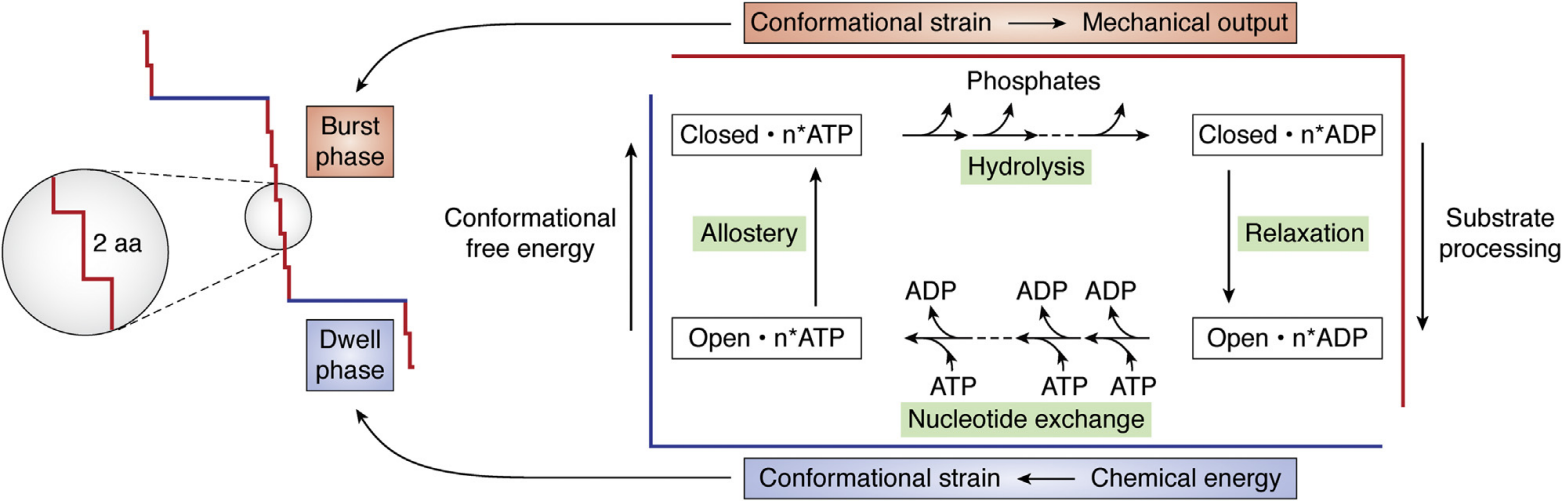
**A cyclic functional strategy.** The HX MS, cryo-EM, and OT results described here outline a cyclic model that alternates between global open-like/NEx and closed-like/SPr structural states, integrated with an energetic ATP binding and hydrolysis cycle. In the initial energy input stage (*blue*), favorable ADP release followed by ATP binding (*bottom*) drive an energetically uphill whole molecule open to closed transition (*left*), priming the physical trigger. In the closed state (*red*), ATP hydrolysis and γ-PO_4_ release (*top*) trigger downhill structural relaxation (*right*), which can be engineered to drive diverse substrate processing outputs. The cyclic model pictures that AAA+ proteins act as an adaptor that converts the chemical energy of cytosolic ATP into mechanical form and then couples it to forceful substrate processing. The nucleotide exchange reset function at the energy input stage and the downhill relaxation-dependent drive at the output stage might be common to AAA+ proteins broadly, whereas the output substrate processing machinery must vary in detail depending on substrate and target system requirements.

The present analysis of HX MS data together with related information defines a series of structural and energetic events that escort Hsp104, and suggestively other AAA+ proteins, around a functional mechanochemical cycle as diagrammed in Figure 4. The intrinsic role of the open state, absent in prior considerations, points to a strategy that separates the active cycle into two alternating structural states designed to perform different functions (blue and red in Fig. 4).

The looser lower-energy open/NEx state is responsible for the nucleotide exchange function. It allows for much faster release of ADPs produced in prior ATPase reactions than could be reached in the tight closed/SPr state. The same property serves the ATP-binding energy-input function. As ADPs leave from the open/NEx state, stochastically in no constrained order (shown by their single exponential kinetics in Fig. 2*C*), they are promptly replaced with incoming ATPs. This step connects the molecular cycle to the external cellular energy supply. The chemical energy of cytosolic ATP is converted into mechanostructural form *via* an allosteric structure change driven by ATP binding.

Neither substrate translocation nor the overall cycle is driven by ATP hydrolysis. Strongly favorable ATP binding to the open/NEx state builds up strain energy and serves to bias the hexameric equilibrium back to the higher-energy closed/SPr state. The closed/SPr state is more tightly structured, coordinating the binding and catalytic sites in preparation for ATP hydrolysis, tightening the pore loops in preparation for gripping and pulling substrate, and poising the protein in a high-energy metastable condition primed to power substrate processing. ATP hydrolysis and phosphate release permit energetically downhill structural relaxation, which is engineered to drive output energy-requiring substrate translocation. Possible stepwise translocation modes have been copiously inferred from cryo-EM image analysis and other information but the details of structure/energy coupling are unknown. Sufficient ATP hydrolysis permits global relaxation back to the low-energy open/NEx state, terminating the closed/SPr state burst phase and reinitiating the open/NEx state dwell period where the release of accumulated ADP and ATP replenishment resets the cycle.

The number of steps in a run of burst phase processing and the power output is limited by the number of ATPs previously bound in the open state. Just as ADP dissociation from the loose open state is stochastic (Fig. 2*C*), ATP rebinding seems likely to be flexible in number and distribution, providing a degree of freedom that is able to affect subsequent substrate processing, possibly relating to the observed irregularities in translocation behavior noted before (44, 45, 46, 47, 48). The hydrolysis of more than one previously bound ATPs in rapid succession can sum to a larger energy drop and a more powerful stroke. Examples include the ability of some AAA+ proteins to adapt to more resistant substrates (16, 54, 55) and the single stroke sundering of the membrane-bound SNARE complex by the nsf (N-ethylmaleimide-sensitive fusion) protein (55). The hydrolysis of sufficient ATP allows transition to the open state. The obligatory return to the loose open state on each cycle can provide a tactic for jettisoning a particularly resistant substrate, avoiding protein stalling and continued wasteful hydrolysis (56). Low cytosolic ADP favors product ADP dissociation, clearing the system for the rebinding of solution ATP in preparation for another round of processing.

Remarkably, each sequential forward step around the functional cycle is energetically downhill (ADP off, ATP on, open to closed, ATP hydrolysis, phosphate release, closed to open). This feature drives the system unidirectionally around the cycle, providing the basis for unidirectional substrate translocation.

## Generalization

The authenticity of the two-state model and its value can be tested by its ability to explain observations in the literature. The model unifies cryo-EM, HX MS, and OT observations. It organizes information that reveals the structural basis of optical trap kinetic phases, accounts for a major cryo-EM population previously largely ignored, reveals the separate roles of ATP binding and hydrolysis, and describes how the energy of cytoplasmic ATP is transduced into mechanically useful form. The model explains the unidirectional transport of substrate, the auto-adjustment of variable translocation force, and the origin of irregularities therein. It depicts the recurrent opportunity to disengage substrate, which would be useful for limited disaggregase activity and for jettisoning intractable substrate.

The present comparison of disparate AAA+ molecular machines suggests that the functional strategy just described is widely distributed. The structural conservation of this cyclic energy input, conversion, and output strategy would enable the core AAA+ motif to serve as a general adaptor to transduce the chemical energy of cytosolic ATP into mechanical form and then into a nearly unlimited range of physical operations. The engineering details that shape substrate engagement and output coupling can freely evolve in the usual way to match the requirements of different target systems, and detailed kinetic parameters may be adjusted, but the essential elements of the energy input/output conversion strategy just described may be unalterable. It seems to us not unlikely that a similar strategy may be conserved even in distantly related systems.

## Conflict of interest

The authors declare that they have no conflicts of interest with the contents of this article.
