# Correction: A conserved strategy for structure change and energy transduction in Hsp104 and other AAA+ protein motors

**DOI:** 10.1016/j.jbc.2021.101285

**Published:** 2021-10-14

**Authors:** Xiang Ye, Leland Mayne, S. Walter Englander

The above review included an error on the top of page six under the heading “Optical tweezers burst and dwell phases represent the closed/SPr and open/NEx states.” The word “open” was used when “closed” was intended. The corrected sentence should read: “The OT observation that substrate translocation occurs in the burst phase associates the OT kinetic burst phase with the cryo-EM structural closed state.”

